# A Critical Review of Existing Test-Methods for External Sulfate Attack

**DOI:** 10.3390/ma15217554

**Published:** 2022-10-27

**Authors:** Mike Jabbour, Othman Omikrine Metalssi, Marc Quiertant, Véronique Baroghel-Bouny

**Affiliations:** 1 Laboratoire Matériaux pour une Construction Durable (UMR MCD), University Gustave Eiffel, Cerema, F-77454 Marne-la-Vallée, France; 2 Laboratoire Expérimentation et Modélisation pour le Génie Civil et Urbain (EMGCU), Materials and Structures (MAST) Department, University Gustave Eiffel, F-77454 Marne-la-Vallée, France

**Keywords:** external sulfate attack, ettringite formation, physico-chemical mechanisms, standardized and non-standardized methods, strengths and weaknesses of each testing method, recommendations for improving methods

## Abstract

External sulfate attack (ESA) of cementitious materials has been studied worldwide for a very long time. This physical/chemical interaction between sulfate ions and the cement hardened elements affects the long-term durability of concrete structures: cracking, spalling or strength loss of concrete structures. To study these damaging phenomena, some standardized and non-standardized accelerated aging tests are used to evaluate the performance of cements in sulfate-rich environments. However, these existing methods do not adequately predict field performance and some shortcomings or deficiencies still exist: change of degradation mechanisms when using high concentrations of sulfate, variable boundary conditions and small specimens compared to the real concrete structures. In this work, a critical review of some existing test methods and foreign national standard methods for ESA are presented, analyzed, and discussed. This results in some proposed recommendations for improving these methods to meet the needs of structure managers.

## 1. Introduction

External sulfate attack (ESA) is a complex concrete structural deterioration process that is initiated when sulfate present in the external environment penetrates into concrete [1,2,3,4,5]. Damage induced by ESA is due to (i) chemical attack, where chemical reactions between the sulfate ions and the cement hardened elements lead to expansion caused by the precipitation of ettringite and/or gypsum and (ii) physical attack, where cracking, caused by expansion and internal stresses, increases the concrete permeability, consequently making this material more vulnerable to the diffusion of aggressive compounds [6,7,8,9,10,11]. The physical attack also concerns the adsorption of free sulfate ions on the C-S-H particles. Despite the large number of publications and research related to the ESA process, there are still some open questions and outstanding issues especially regarding the mechanisms involved in this reaction.

Indeed, a large part of the complexity of this reaction stems from the variety of ions like calcium, sodium and magnesium sulfate that can be involved in the alteration of the paste composition [12]. Moreover, in the case of sodium sulfate, the ability of gypsum to trigger a significant increase in volume and to damage the cement paste is still debatable and remains questionable [6,7,12,13]. Some studies have stated that the gypsum formation during the sodium sulfate attack is the principal mechanism contributing to surface deterioration and a decrease in the mechanical resistance [14]. However, it is believed that gypsum can occupy the available air voids in the cement paste; subsequently, it initiates volumetric swelling [15,16,17,18,19,20,21].

Although the mechanisms associated with ESA remains a highly active and controversial research area, there is a need to build concrete infrastructures even in places where concrete is exposed to a sulfate-containing environment. In this context, the industry has outlined several blended cements with improved performance against ESA and several laboratory test methods have been developed to determine the sulfate resistance of these cement mixtures.

Usual ESA testing methods involve the immersion of cement paste, mortar or concrete samples into a sulfate solution leading to the diffusion of sulfate ions through the samples. After several months to more than one year of immersion, such tests can be used to assess the change in the lengths and masses of samples induced by ESA in parallel through visual inspection [12,22,23,24,25,26]. However, several issues related to acceleration methods have been noted. ESA in the field is a slow process taking decades to produce damage, whereas the acceleration of the attack in the laboratory is achieved by (i) using high sulfate solution concentrations (e.g., >30 g/L) to accelerate the sulfate ingress, (ii) storing samples at high temperature (e.g., >30 °C) or low temperatures (e.g., <15 °C) to promote the reaction kinetics and (iii) reducing the curing time and increasing the w/c ratio to produce a more porous material, leading to an increase in the transport process [27]. The representativeness of the attack mechanisms experienced during the accelerated tests is then questioned, not only due to their faster kinetics but also considering, for example, that the mechanism of the ESA changes with the solution concentration. According to results produced by Biczok [28], ettringite is the major product that accumulates at low sulfate concentrations and gypsum is the major product at high sulfate concentrations.

Moreover, the two accelerating methods of exposing samples to a high sulfate solution and high temperature do not represent the real conditions: the high concentration commonly used in the accelerated tests corresponds to about ten times the maximum values found in the field, and this could change the degradation mechanisms occurring in the concrete structures. Furthermore, the typical water temperature in the field range from 10 to 20 °C, which is significantly lower than that maintained during accelerated tests [7,12,22,23,24,28].

In contrast to the lack of representativeness of the previous acceleration protocols, it is notable that the reduction in the curing time of a sample as an accelerating tool can correspond to a representative field condition considering that concrete structures can be exposed to external aggressive environments early in their construction. This early-age exposure has an obvious influence on the concrete strength and transfer properties, which are first-order parameters affecting the durability of the material.

Current test methods have been criticized in several publications for their slowness or exposure conditions, such as pH and sulfate amount in immersion solutions [7,13,29,30,31,32]. Indeed, these parameters may change over time if the solution is not periodically renewed [27]. For example, the leaching process changes the pH of the immersion solution from neutral to about 12.5 (equilibrium pH of the pore solution). These later exposure conditions are therefore less severe for the samples and lengthen the duration of the test in comparison to a test that maintains the concentration over time [27,32,33,34,35,36,37,38,39,40]. Furthermore, the change in the exposure conditions does not represent the in-situ reality when it is considered that the reactants are inexhaustible and always accessible.

Accelerated test methods for evaluating the properties of cementitious materials in sulfate-rich environments such as the ASTM method [41] have received several criticisms (i) the cause of expansion is not determined by the tests, (ii) the addition of sulfate and inadequate curing are not representative of field conditions and (iii) the pH evolution during the test is not representative of the field conditions [13,23,33,42,43,44]. Consequently, many researchers consider these test methods insufficient for predicting field performance [30]. To address this issue some authors suggest that the pH of the solution should be maintained at a constant value (e.g., pH = 8 for seawater) with periodic renewal of the sulfate solution [16,31,44,45,46,47]. Maintaining a constant pH helps to overcome some problems associated with ASTM methods [41] especially those related to representative field exposures and those that significantly increase the rate of sulfate attack [16,27]. Additionally, the control of this parameter is more likely to occur in real environments. Likewise, pH conditions can affect the mechanism of the ESA. The pH value can reach 12.5 for a few hours due to the calcium leaching from portlandite and then, the ESA leads to the formation of ettringite, which causes the expansion of the concrete samples [16,43]. However, when the pH value is between 8 and 11.5, ESA allows gypsum formation and C-S-H decomposition resulting in reduced cohesion [48]. Brown’s [29,30] study showed that the expansion rate increases when the pH of the immersion solution is kept below 11.5. On the other hand, most ESA accelerated tests have been performed under non-representative field conditions (e.g., 50 g/L for most sulfate concentration testing protocols). However, in seawater the sulfate concentration is around 2.7 g/L while in the groundwater or soils, this value can be much lower.

There are several review papers in the literature of accelerated test methods applied to the study of ESA [13,49,50,51,52,53]. Therefore, the purpose of this paper is to present the main testing methods used in laboratories, discuss their advantages and disadvantages, and present some examples of research results obtained by these testing methods. This critical review can help select a particular method tailored to a particular study or develop an innovative rapid test tailored to the needs of a particular research project.

This paper is organized as such: after this introduction, Section 2 introduces and describes existing standard and non-standard experimental methods with some examples of experimental frameworks used to evaluate the performance of cementitious materials against ESA. Section 3 discusses the strengths and weaknesses of each testing method with the opinions of the authors and finally some conclusions and recommendations are drawn.

## 2. Overview of the Existing Test Methods

Several tests are available to study the different aspects of ESA. Some of the experimental methods replicate the process of this attack by immersing the samples in a test solution or by performing continuous wetting and drying cycles [23,33,54,55,56]. There are several types of sulfate solutions (sodium, calcium and magnesium sulfate) and the compounds and concentrations used depend on the needs of the research. In several studies [23,33,55], the samples were immersed in the same test solution throughout the entire accelerated ageing period, but in some other studies, the solution is periodically renewed [29,30]. Some parameters of the samples may also vary, depending on the applied experimental protocol, like the shape (prismatic or cylindrical) and size of the samples or the material under study (concrete, mortar or cement paste) or its w/c ratio.

In a previous overview [48] an increase in the diameter of cylindrical concrete samples was used as the expansion indicator of the sulfate resistance. Hughes [57] tried to study the effects of curing, permeability and pore size distribution on the sulfate resistance of cement materials using three types of cement pastes and 0.47 w/c ratio (i) Ordinary Portland Cement (OPC), (ii) OPC with 30% fly ash and (iii) OPC with 0.5% C3A. Three samples were used for each test to obtain an average value and standard deviation. Three curing durations were considered (1, 4 and 12 weeks) for both OPC and OPC/fly ash pastes. Curing was performed by full immersion of samples in a Ca(OH)_2_ solution at 35 °C. After curing, the samples were immersed in a sodium sulfate solution (0.7 M) at 25 °C for 60 days. Hughes [57] evaluated the variation in expansion, permeability and pore size in correlation with the curing duration in order to explain the resistance mechanisms used by the tested mixtures against ESA. According to the experimental observations, it seems that increasing the duration of the curing process before initiating the attack increased the resistance against ESA, especially for cement containing fly ashes (OPC with 30% fly ash). However, the ability of OPC samples to resist sulfate diffusion was found to be independent regardless of curing time. Furthermore, Hughes [57] stated that both the level of permeability (related to the diffusion kinetic of sulfate ions in the material) and the size of the pores occupied once sulfate ions have penetrated, can affect the resistance against ESA.

All of the different parameters considered in the experimental approaches affect the duration of each test method. Duration varies from weeks to months. Recently, the use of a continuous pH test titration method has enabled the exploration of new features and indicators related to resistance to ESA [6,15,58,59]. In these studies, the specimens’ deterioration was characterized by mass loss, expansion variation and change in radius and/or surface roughness. Change in the compressive strength and elasticity modulus are also used as important indicators. In addition to the above-mentioned test methods, other approaches use techniques like microstructural analysis and chemical analysis (NMR, SEM/EDX; XRD and XRF) to study the formation of ettringite, gypsum and thaumasite in the pores of the attacked specimens [60,61,62,63]. Moreover, other techniques are used by researchers to obtain further information on the progress of ESA on cementitious materials [46,59]. For example, ion chromatography (IC) as well as pH and conductivity measurements were used to evaluate sulfate adsorption and the total amount of adsorbed sulfates respectively [15,43,58,59]. Additionally, there are several pulse stimulation-based techniques to study changes in E-modulus that can indicate ESA-induced damage [64,65,66,67].

In the following text, a review of current test standards or research methods used for testing the performance of cementitious materials subject to ESA is presented and discussed. Moreover, some relevant case studies using the presented test methods are also reported that use the presented test methods to clarify and enrich the discussion.

### 2.1. Standardized Methods

#### 2.1.1. ASTM C 452-15

ASTM C 452-15 [41] suggests an experimental method to accelerate the ESA-induced expansion of mortar bars that involves the addition of sufficient gypsum to the dry OPC prior to mixing. In this way, the mixture contains around 7% of the mixture of sulfur trioxide (SO_3_) by mass, which leads to internal formation of ettringite without external sulfates’ penetration. This method is not suitable for establishing the sulfate resistance of cement mixed in combination with pozzolans or slag. The mortar samples used in this technique are 25 mm × 25 mm × 285 mm prisms. Each specimen contains stainless-steel gauge studs inserted into its ends which help to detect the length changes during the attack. Mortar samples are cured in specific molds for 23 h, demolded, and have their lengths measured before being immersed in deionized water. After 14 days, the longitudinal expansion of each sample is measured [34,54,55,56,66]. Due to this short immersion duration, the test is recognized as one of the fastest to assess the mortar’s resistance to the sulfate attack. In this method, after 14 days, the range of expansion does not exceed 0.04%. For many researchers, the sulfate durability tests proposed in ASTM C 452-15 are considered to be effective for differentiating high C_3_A and low C_3_A Portland cement according to their levels of sulfate susceptibility. However, they are not considered applicable for blended cements. This can be explained by the fact that compound binders require a curing time of longer than 14 days for sufficient hydration. The test prescribed in ASTM C 452-15 [41] can result in a sulfate attack of anhydrous compounds that does not correspond to chemical processes occurring in the field. In addition, the presence of gypsum seems to cause the sulfate attack in the fresh state. This early attack means that the testing conditions of ASTM C 452-15 are not similar to the field exposure of concrete to sulfate, which involves the ingress of sulfates into the concrete [15,54,55,56,57,58,66,67,68,69].

**Figure 1:**
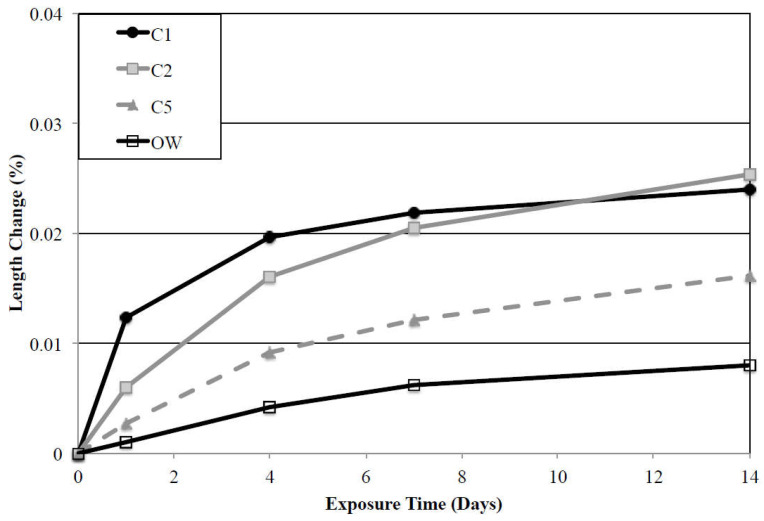
Length change over time for different mortars tested using the ASTM C 452 method [15].

Figure 1 presents experimental results obtained by Aguayo [15] while studying the expansion of four types of blended mortar mixtures measured after 14 days of immersion in deionized water, where the length change was evaluated using the procedures described by the ASTM C 452-15 method. Three types of cements with varying tricalcium aluminate (C_3_A) contents were studied: type I cement (C1), type I/II cement (C2), and type V cement (C5) with C_3_A contents of 11.52%, 6.80% and 3.80% respectively. Moreover, a class H oil well cement (OW), with approximately no C_3_A content was also studied. The method was found to be an efficient way to correlate the expansion rates of mortar samples with the C_3_A content. Finally, this correlation was interpreted as an indicator of the cement type resistance against ESA. From the results, it is clear that sample types C1 and C2 showed the greatest length changes (0.024% and 0.025%, respectively) after 14 days in contact with deionized water. However, mortar samples blended with cement types V and OW showed overall expansion rates of 0.016% and 0.008%, respectively. The differences among the four types of studied mortar samples are related to the presence of additional anhydrous particles in type C1 and C2 cements with higher C_3_A contents [15]. The results shown in Figure 1 highlight the effect of C_3_A anhydrous cement on the expansion rate of mortars. This confirms that this anhydrous compound is a source of additional aluminates that react with gypsum and sulfate ions to produce the secondary ettringite responsible of the mortar expansion.

#### 2.1.2. ASTM C 452-15

**Figure 2:**
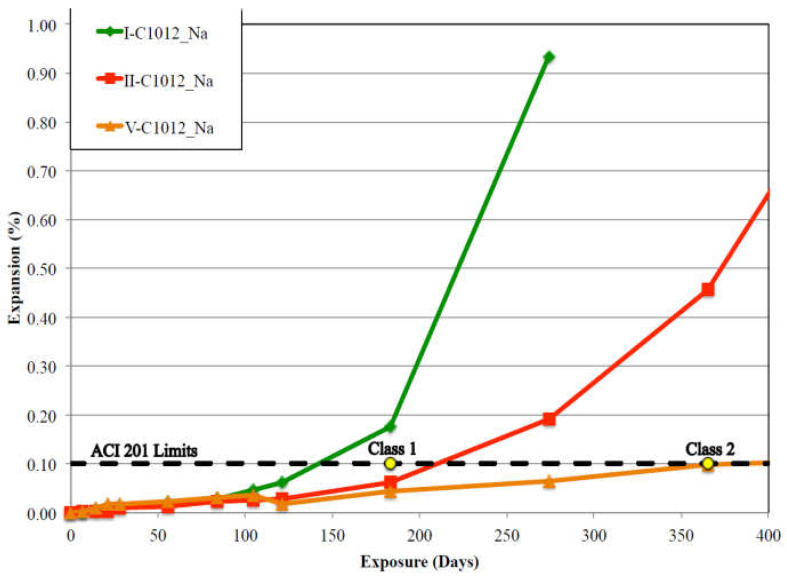
Expansion evolution of different cement-based materials over time using the ASTM C 1012 method [15].

Due to the limitations imposed by the ASTM C 452-15 [41], the ASTM C 1012/C 1012M-18b test [70] was developed to be applicable to cement formulations containing mineral additions and then to assess binder’s resistance to sulfate attack. Even though this test is not considered to be the best or the most connected to real-life exposure conditions, it has been used by many researchers to evaluate the sulfate resistance of cement-based materials [69,71]. The experimental procedure involves the use of a mortar mixture cast with 1 part cementitious material (Portland cement, blends of Portland cement with pozzolans or slags, and blended hydraulic cements) to 2.75 parts sand by mass. The w/c ratio is fixed at 0.485 for all non-air-entraining Portland cements and 0.460 for all air-entraining Portland cements and for mixtures incorporating supplementary cementing materials (SCMs); water is added in order to reach a flow rate of within ±5 of the flow of the control mixture (plain Portland cement mortars with a 0.485 w/c ratio). Samples used in this method are 25 mm × 25 mm × 285 mm mortar prisms with stainless-steel gauge studs embedded into the ends for length measurements [69,71]. In addition, 50 mm^3^ mortar cubes tested for strength were cast from each mixture. The curing process involves placing all types of samples into a 35 °C ± 3 °C water bath for 23 h ± 0.5 h. After curing, the specimens are demolded, and two cubes are tested for compressive strength. Once the average compressive strength of cubes has reached 20 MPa, the mortar bars are then placed into a 5% sodium sulfate solution. Upon immersion in the sulfate solution, expansion measurements are taken at pre-determined time intervals: after 1, 2, 3, 4, 8, 13, and 15 weeks and then 4, 6, 9, 12, 18 months thereafter [15,54,55,56,57,58,59,69]. To be considered sulfate-resistant, the specifications of this test imply a specimen expansion limit of 0.05% based on the type of cement used in the mix after 180 days of immersion [15,34,66]. The results obtained from this technique show that even though ASTM C 1012 is a much longer test method compared to ASTM C 452, it can still be considered an efficient approach to assess the sulfate resistance of mortars made using Portland cement, blended cements designed to be used in combination with pozzolans or slags, and blended hydraulic cements produced by blending two or more types of fine material (Portland cement mixed with either blast-furnace slag, fly ash, silica fume or other pozzolans). For example, Figure 2 illustrates the results presented in the dissertation of Aguayo [15] who evaluated the rate of expansion induced by sodium sulfate attack by applying the ASTM C 1012 method for the three types of mortar previously used in the ASTM C 452 method (type I (C1), type I/II (C2) and type V (C5) cements with C_3_A contents of 11.52%, 6.80%, and 3.80%, respectively). From the results presented in Figure 2, it is clear that the C_3_A content has a significant effect on the performance of cementitious mixtures against ESA [15]. Type I cement exhibited the lowest resistance to ESA with a percentage of expansion exceeding the ACI 201 [72] limit of 0.1% after one year of exposure. However, mortar samples mixed with type C5 cement showed an expansion level of approximately 0.1% after 350 days of exposure. The results illustrate the effect of a low C_3_A content in terms of the increase in the resistance against ESA. Finally, by comparing the results shown in Figure 1 and Figure 2, it is clearly highlighted that even if the ASTM C 1012 method takes more time (more than one year), it leads to characterize the expansion kinetics as the swelling curves can be divided into two parts: the latency part until around 180 days and the characteristic part after this time until the complete damage of the samples (Figure 2). However, the ASTM C 452 method gives just an idea of the expansion behavior of the mortars during the first days with very small swelling rate (Figure 1).

#### 2.1.3. USBR 4908

Instead of conducting sulfate resistance tests on mortar bars as specified in ASTM C 452 [41] and ASTM C 1012/C1012M-18b [70], the United States Bureau of Reclamation recommends the use of the standardized test USBR 4908 [73] for researchers interested in testing actual concrete samples in order to achieve a better representativeness of the service conditions. The USBR 4908 method is similar to the ASTM C1012 regarding the applied conditions. However, the main difference lies in the types of specimens that are made of concrete and have a cylindrical shape: 150 mm in diameter and 300 mm in height (6 in and 12 in) [70,74]. In this method, the specimens are immersed in a sulfate solution with two different concentrations (2.1% and 10%) of sodium sulfate. The method is applicable to concrete mixes containing Portland cements, blended cements, or blends of cement and mineral admixtures. USBR 4908 has a significant flexibility as it is able to evaluate different aspects related to the permeability of tested samples, mineral and chemical admixtures as well as various curing methods [15]. USBR 4908 provides three different experimental approaches in which the cylindrical specimens are exposed to a sulfate solution:

- In the first approach, the samples are immersed in a 2.1% sodium sulfate solution;
- In the second approach, the samples are immersed in a 10% sodium sulfate solution;
- In the third approach, the samples are subjected to wetting/drying cycles consisting of 16 h immersion in a 2.1% sodium sulfate solution and 8 h of drying under forced air at 54 °C.

**Figure 3:**
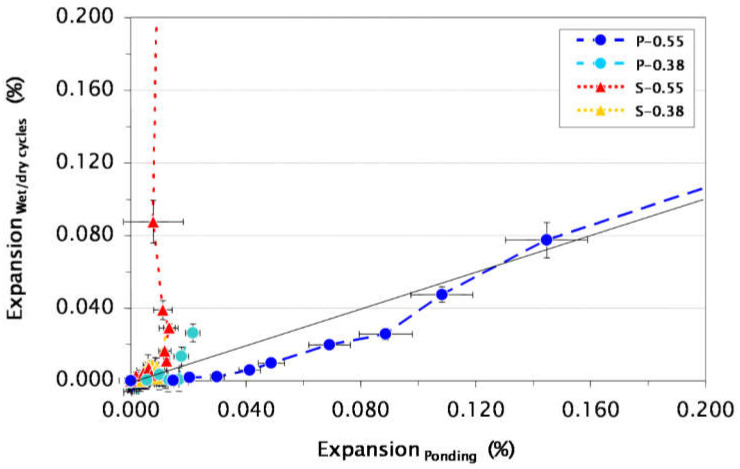
Expansion under wet/dry cycles versus expansion under full immersion of different micro-concrete samples [27]. The black line is the regression line (relationship between the expansions of P-0.55 in ponding and wet/dry cycles) proposed by the author.

A test period of at least 1 to 2 years is generally required before any significant expansion and mass variations may be obtained, which means that this test duration is not suitable for supporting the continuous development of new sulfate-resistant cements. As seen in Figure 3 [27], the expansion evolution of cement-based materials differs between full immersion and wet/dry cycles. In this experimental program, four micro-concrete sample types with two w/c ratios were tested. Samples P-0.55 and P-0.38 were mixed with CEM I cements with two different w/c ratios (0.55 and 0.38, respectively). On the other hand, samples S-0.55 and S-0.38 were mixed with CEM III/B cement and with the two previous w/c ratios (0.55 and 0.38, respectively). The exposure conditions used in this study were inspired by the USBR 4908 approach with a difference in the concentration of sulfate solution (3 g/L). The results shown in Figure 3 indicate that samples mixed with CEM III/B cement are somehow more sensitive to continuous wet/dry cycles with respect to expansion. However, samples made with CEM I show a rate of expansion that can be comparable between wet/dry cycles and full immersion. In addition, the results show that the evolution in the rate of expansion for samples type P-0.55 (CEM I with w/c = 0.55) follows a progression line (x = 1.75y). Thus, it can be noted from this experimental work that slag blends have less resistance with exposure to wet/dry cycles, which is not the case for CEM I mixes, which have similar behavior in both ponding and wet/dry cycles [27].

Many publications indicate that USBR 4908 has the ability to evaluate the effects of several characteristics of this material on the sulfate resistance of concrete, including the permeability, mineral and chemical admixture contents, and other mix design alternatives, as well as the effects of various curing procedures (see for example [6,8,16,27,41,45,46,66,69,71,73,75,76,77,78]).

**Figure 4:**
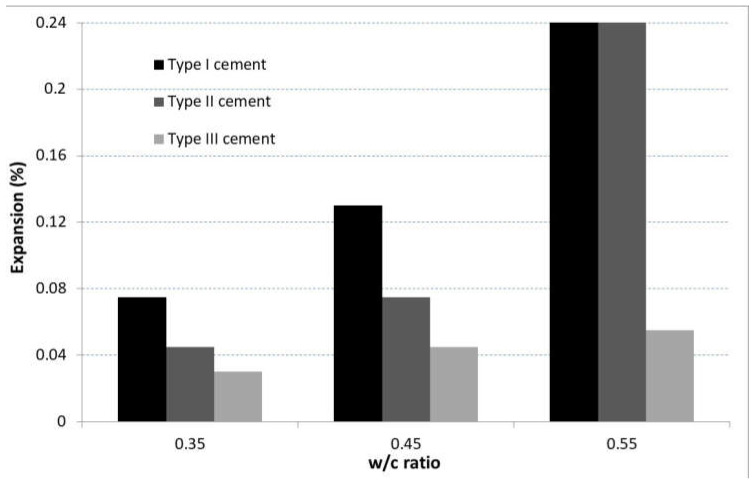
The expected expansion rates obtained by ANN after two years of immersion in a 10% sodium sulfate solution (from [79]).

In a study by Hodhod and Salama [79], the authors applied the second accelerating approach listed in the USBR 4908 [73]. Then, they immersed concrete cylinders (76 mm in diameter and 152 mm in height) in a 10% sodium sulfate solution for a period of 6 months. The study evaluated the performance levels of different types of cements (Type I Portland cement, Type II cement and Type V cement) mixed with three different w/c ratios (0.35, 0.45 and 0.55). The sulfate-attack-induced expansion response of the tested mixes after two years of immersion in the sodium sulfate solution was predicted with an artificial neural network system (ANN). The results illustrated in Figure 4 show that Type I cement exhibited the greatest expansion of all w/c ratios. However, Type-V-based concrete cylinders did not show significant expansion. This behavior was attributed to the low C_3_A content (5%) of their constitutive cement. Interestingly, the highest expansion level reached by each type of sample was obtained for cylinders mixed with a w/c ratio of 0.55. These findings obtained by the ANN model prove that both the water and C_3_A contents affect the response of concrete to ESA [79].

#### 2.1.4. The European Committee for Standardization (CEN)

The European Committee for Standardization (CEN) does not prescribe a standard test method for evaluating the sulfate resistance of a Portland or blended cement. Despite many European countries having their own testing techniques and recommendations, the development of a unified European standard for sulfate-resisting cements has not been accomplished and remains as a debatable topic due to national differences in the types of cement recognized as sulfate resistant [15,66,80]. However, the CEN published a technical report [80], outlining the current status of sulfate resistance. The report gathers important information from over 100 papers and publications that were available from an access database. This step was needed in order to analyze the different sulfate resistance testing techniques and their possible influences on the assumed performance of different binders used in Europe [16,46,58,59,66].

#### 2.1.5. Swiss Standard SN 505262-1

**Figure 5:**
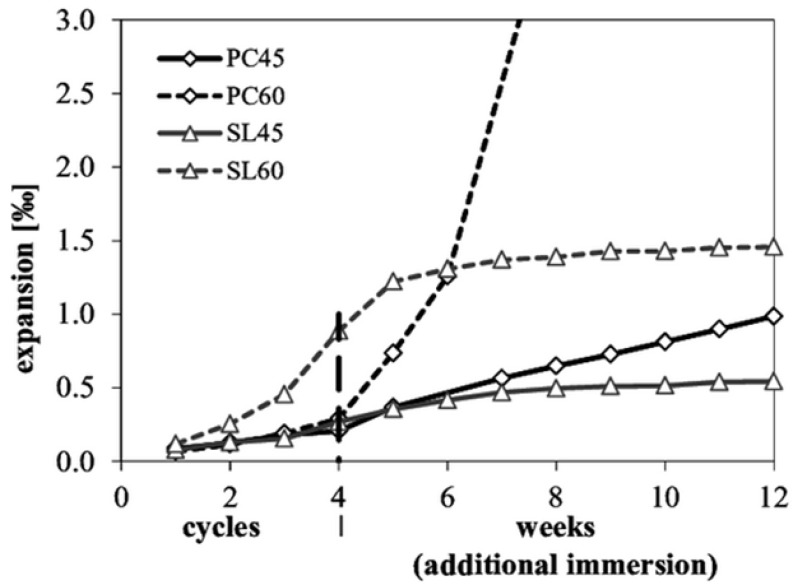
Expansion of the four types of concrete samples tested by Leemann and Loser [82].

In connection with the two “Alp transit tunnel” projects conducted in Switzerland, an accelerated test using drying–immersion cycles to accelerate sulfate ingress was developed based on the Swiss standard [81]. In the experimental work of Leemann and Loser [82], the microstructural and chemical aspects of the sulfate attack on various types of concrete samples were analyzed based on the expansion variation as well as the sulfate distribution within the attacked specimens. Two w/c ratios (0.45 and 0.60) and two cement types (CEM I/OPC and CEM III/B) were considered for the mixture design of samples. The reference samples were mixed using OPC, while other samples were made of industrially mixed slag cement containing 20–34% OPC and 66–80% blast furnace slag considered to be high-sulfate-resistant cement (CEM III/B). Immediately after casting, the 150 mm^3^ concrete cubes were stored at 20 °C and 90% of RH for 24 h. Once demolded, the specimens were stored for 26 days under the same climatic conditions. After this curing period, seven concrete cylinders (28 mm in diameter and 148 mm in height) were cored from each concrete cube with a plug gauge bonded at their extremities in order to monitor the strain. The sulfate resistance of the different 28-day-old specimens was evaluated based on the Swiss standard SN 505262/1 [81,82]. This method differs from the traditional techniques encountered in the literature due to the inclusion of curing and drying approaches where specimens are subjected to an accelerated attack consisting of four consecutive cycles of drying inside an oven at 50 °C for 120 ± 2 h, followed directly by a full immersion phase in a 5% sodium sulfate solution for a period of 48 ± 2 h. At the end of these sequences, the concrete specimens were fully immersed in the previously used attacking solution for an additional period of two months. By comparing the expansion of concrete samples fabricated with OPC (PC45 with a w/c ratio of 0.45 and PC60 with a w/c ratio of 0.60) and the ones made with mineral additions (SL45 with w/c ratio of 0.45 and SL60 with w/c ratio of 0.60) (Figure 5), it was observed that sample SL60 had the most significant expansion (0.9‰) at the end of the drying–immersion cycles, while samples PC45, PC60 and SL45 showed considerably lower expansion rates of 0.2‰, 0.25‰, and 0.21‰, respectively [82]. During the additional immersion phase, a noticeable shift in the results were seen with sample PC60, which exhibited a major and fast increase in expansion (>3‰). The same behavior was observed for samples PC45 and SL45 with increases in expansion from 0.2‰ to 0.8‰ and 0.21‰ to 0.5‰, respectively. The length variation measurements show that the higher the w/c ratio is, the greater the expansion is. Furthermore, the type of accelerated attack can heavily determine the level of damage done to concrete, regardless of the mixture used. For example, the previously discussed experimental work showed that concrete samples with OPC were more sensitive to full immersion in a sodium sulfate solution, in contrast to samples with CEM III which expanded more during the drying–immersion cycles.

#### 2.1.6. Canadian Standard (CAN/CSA-A3004-13)

**Figure 6:**
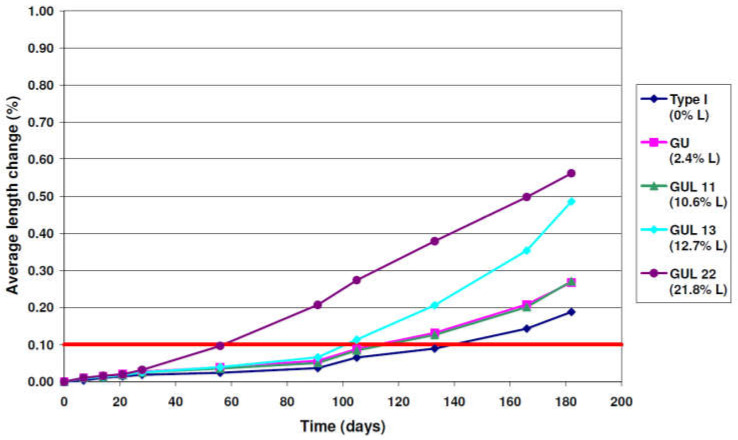
Expansion of mortar prisms stored at 23 °C from [84]. Red line marks the expansion limit of 0.1%.

The testing method proposed in the Canadian standard to determine the sulfate resistance of blended cement mortar samples against ESA is described in CSA A3004-13 [83]. This method is very similar to the ASTM C 1012 method. Indeed, both the American and Canadian standards specify the same limits for the C_3_A content and for the maximum allowed percentage of expansion induced by ESA. However, the CSA A3004-C8 [83] testing method differs as it considers two procedures, thus enabling both the ettringite and thaumasite forms of the sulfate attack to be considered. The first procedure was the same as that found in ASTM C 1012/C1012M-18 [70], where mortar prisms (25 mm × 25 mm × 250 mm) were immersed in a sulfate solution (50 g/L) at 23 °C. The main purpose of this procedure was to analyze the ettringite formation induced by ESA. The second procedure involved the same process but with different storage conditions (5 °C). This low temperature helped us to study the specific effects induced by the formation of thaumasite due to sulfate exposure. In both procedures, the duration of the accelerated attack varied between 12 and18 months. For example, in his thesis, Ramezanianpour [84] was inspired by the testing method listed in the Canadian standard and used this to study the progression of the sulfate attack using five different types of cement mixtures (Type I: CEM I, GU: CEM I with 2.4% limestone, GUL11: CEM I with 10.6% limestone, GU13: CEM I with 12.7% limestone and GUL22: CEM I with 21.8%). In this experimental program, one cement clinker was used to produce five types of cement with various limestone contents. Some modifications of the CSA A3004 [83] method were introduced by using mortar prisms (25 mm × 25 mm × 285 mm) and cubes (50 mm^3^). All sample types were subjected to drying at 38 °C for 24 h. After this drying period, the samples were demolded and submitted to another cure inside saturated lime water for another 24 h at 23 °C. Firstly, samples were immersed in a 50 g/L sodium sulfate solution at 23 °C with length change measurements conducted before contact with the sulfate solution and at 1, 2, 3, 4, 8, 13 and 15 weeks after the attack. The following part of the test consisted of applying the second procedure of CSA A3004 (low temperature test identified as Procedure B in [83]) in order to evaluate the effects of thaumasite formation. Subsequently, the process used in the first stage of the experiment was maintained (mix proportions, casting, molding and curing) but mortar prisms were stored in a refrigerator for 24 h at 5 °C before being fully immersed in the 50 g/L sodium sulfate solution at 5 °C. The average length change of the samples immersed at 23 °C is shown in Figure 6 [84], where it can be seen that the expansion limit of 0.1% was exceeded by all tested mortars after an immersion duration of less than 6 months with a significant increase for cement type GUL 22 (21.8% limestone). These observations indicate that the expansion increased with an elevated percentage of limestone.

**Figure 7:**
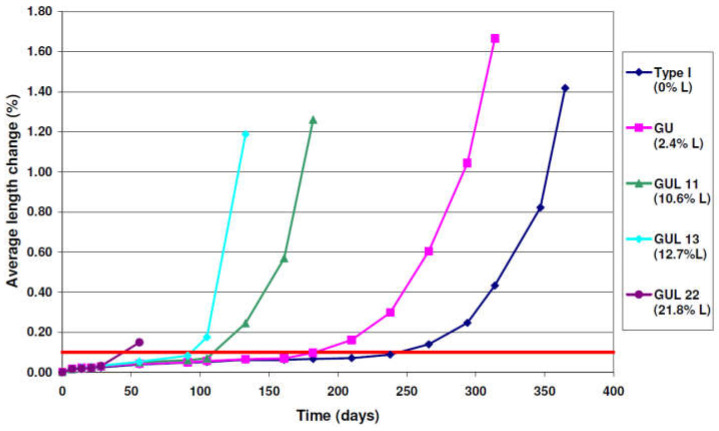
Expansion of mortar prisms stored at 5 °C from [84]. Red line marks the expansion limit of 0.1%.

Moreover, expansion during the accelerated attack at 5 °C (Figure 7) [84] also indicated that all samples reached the expansion limit of 0.1% even before 6 months of immersion. These results (obtained from samples immersed at 5 °C) are similar to the previously described ones (obtained from samples immersed at 23 °C), especially when considering the direct relationship between the expansion and percentage of limestone content in the mixture. For example, the GUL22 sample with the highest percentage of limestone exceeded the expansion limit in a short period of time (45 days) when compared with other samples with smaller limestone contents. These results indicate that the presence of limestone in the mixture can accelerate the damage caused by ESA. Also, the effect of temperature of the exposure solution on the expansion rate was highlighted; at this temperature, the three reaction modes are occurring simultaneously in the mortar (the formation of thaumasite, gypsum and ettringite), compared with 23 °C where only ettringite is formed as the other constituents stay thermodynamically unstable or react with other anhydrous or hydrates to form ettringite. These three modes allow enhancement of the mortar swelling and then damage of the materials. However, the low-temperature 5 °C sulfate resistance test has been deleted from the Canadian standard, as from further research the test was found to be overly aggressive and not representative of sulfate resistance of concrete [85,86,87].

#### 2.1.7. Chinese GB 749-1965, GB/T 2420-1981, GB/T 749-2001, and GB/T 749-2008 Standards

Several Chinese standards were established to prescribe ESA test methods [88,89,90]. The GB 749-1965 standard [88] adopts mortar specimens with a binder-to-sand ratio of 1:3.5 and a rectangular shape of 10 mm × 10 mm × 30 mm. In order to ensure consistent test results, the specimens are press-molded, cured in moisture for 1 day, and then cured in fresh water for 14 days before exposure to a sulfate solution for 6 months. The ESA resistance of cement is expressed by the corrosion coefficient. The corrosion coefficient is the ratio of the flexural strength of the cement mortar specimens immersed in the erosive solution to the flexural strength of the specimens immersed in fresh water of the same age. The evaluation criterion is as follow: when the corrosion coefficient at 6 months is less than 0.80, cement is considered to have poor ESA resistance. The advantage of this method is that it has a clear evaluation criterion. However, this method requires a large number of formed specimens and a long test period. At the same time, this method does not specify the concentration of the etching solution and does not consider the difference in the etching mechanism at high and low concentrations. On the basis of GB 749-1965 [88], the test method of GB/t 2420-1981 [89] was developed. It uses 1:2.5 mortar, 10 mm × 10 mm × 60 mm prismatic specimens, pressure molding, 1 day curing and then conservation in 50 °C water for 7 days prior to exposure to a 2% sulfate solution. Regardless of whether GB/T 749-1965 [88] or GB/T M20-1981 [89] is followed, small-sized cement mortar specimens are used, which cannot fully reflect the sulfate resistance of concrete. More recently, the GB/T 749-2001 [90] test method was developed. This uses the expansion ratio as an index to evaluate the ability of cement to resist ESA.

As described above, GB 749-1965 [88] is used to evaluate the ESA resistance of cement, but its disadvantage is that the test period is long. The duration of GB/t 2420-1981 [89] is shorter, but this method has major disadvantages: the size of the test specimen is small, and there is no proposed complementary analysis method to explain the degradation mechanisms that lead to the test results. Thus, the results obtained from the test can only be used to evaluate the ESA resistance but not to understand physical/chemical kinetics of the ESA. The test methods used by some domestic research institutes are derived from this, and they also have limitations. In the ASTM method (see previous description), the ESA resistance of concrete is measured by expansion, which is inconsistent with the actual working environment of concrete, because the expansion damage done to concrete in a large number of buildings is limited by the environment and load. The expansion in the laboratory test is the expansion value under unconstrained conditions, so it is not appropriate to measure the erosion rate of the constitutive concrete of a considered structure in the field using this expansion rate. A revised concrete sulfate resistance test method was proposed. This can be divided into two categories: the total immersion method and the dry–wet cycle method. The main disadvantage of the full immersion method is that the test period is too long, which is not conducive to laboratory research. In the dry–wet cycle method, the control of temperature changes is mainly used to accelerate the crystalline expansion of aggressive ions inside the concrete, but the value of the optimum temperature for studying the resistance of concrete is unknown. In addition, when using the dry–wet cycle method, there are still some issues, such as determining the most suitable solution for soaking, the optimal soaking concentration, and the optimal soaking duration. These factors need to be studied further.

**Figure 8:**
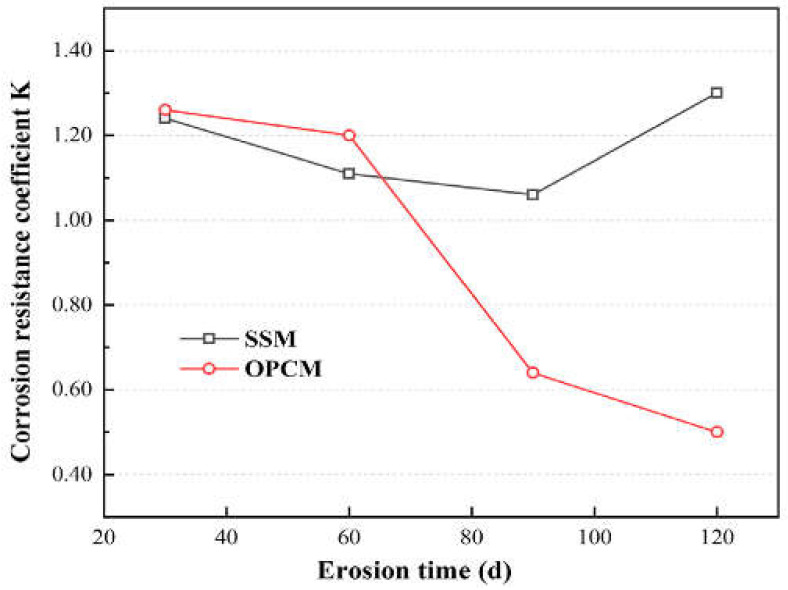
Effect of blended steel slag on the corrosion resistance coefficient k (OPCM: ordinary mortar, SSM: slag mortar) [91].

Finally, there is an update of the GB/T749-2001 test method that was published in 2008 (GB/T749-2008) [90]. In this test standard, the pH of the Na_2_SO_4_ solution should be maintained at around 7 throughout the test which can represent field conditions. Also, the Na_2_SO_4_ solutions were replaced every month to ensure that the experimental conditions are closer to the field conditions, in which mortar is exposed to a continuous supply of sulfate ions. This standard method allows calculation of a corrosion resistance coefficient that evaluates the ability of cementitious materials to resist ESA. An example of the result given by this method is highlighted in Figure 8 [91]. This figure shows that adding the blended steel slag in the mortar mix design (SSM) enhances the corrosion resistance coefficient compared to the ordinary mortar (OPCM).

### 2.2. Non-Standardized Methods

#### 2.2.1. Method of the Laboratory of Materials and Durability of Constructions (LMDC-France)

**Figure 9:**
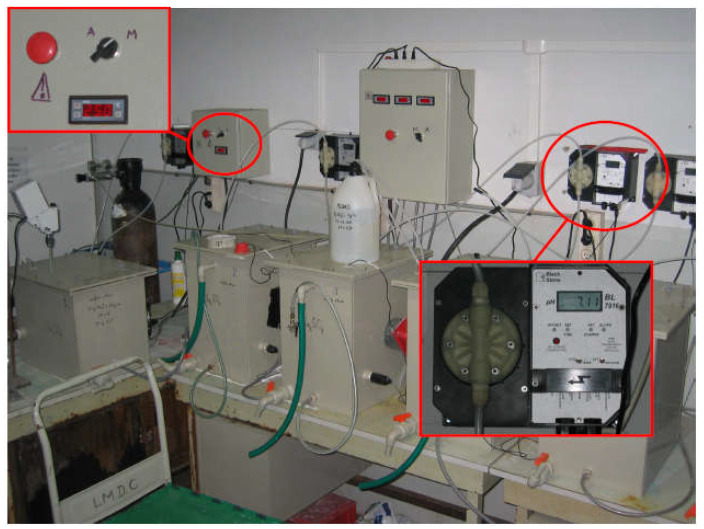
Experimental setup used in the accelerated aging test of Messad with a zoomed in view of the pH regulator [58].

Various experimental works are listed in the literature as ways to accelerate ESA; however, the majority of the testing procedures require a long exposure time. For example, in the current French standard established for special products intended for hydraulic-binder-based concrete work [92], the test operating time used to assess resistance to seawater and sulfated water is considered long, with exposure to magnesium sulfate varying from 6 months to one year before seeing any noticeable sign of degradation. In addition, the cations (Mg^2+^) associated with this test lead to a particular type of ESA that is mostly encountered in marine environments. However, the experimental protocol proposed by Messad [58] uses a high sulfate concentration solution (8.9 g/L of sodium sulfate), a controlled temperature (25 °C), and a fixed pH value (7) to accelerate the ESA. The results from this trial show that, under the proposed conditions, deterioration of the tested concrete samples was observed after a period of only 12 weeks. This short sulfate exposure period is considered one of the main advantages of this protocol in which three parameters are simultaneously controlled during the accelerated attack. The experimental setup designed for the accelerated tests is illustrated in Figure 9, and the experimental conditions used by Messad [58] are listed in Table 1.

**Figure 10:**
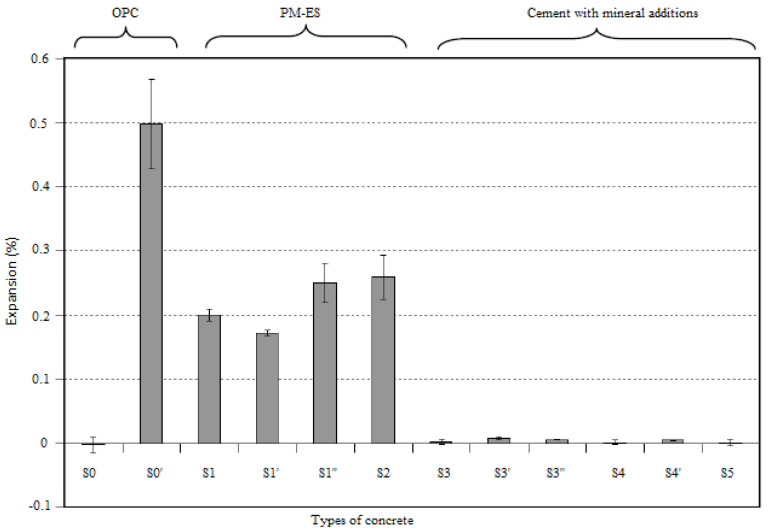
Expansion after 12 weeks of accelerated sulfate attack for all concrete sample types used in the study by Messad [58]. See [58] for details of the twelve concrete mixes (S0–S5).

In the protocol proposed by Messad, both the cylindrical (110 mm in diameter and 220 mm in height) and prismatic (7 cm × 7 cm × 28 cm) specimens were demolded and placed directly in water for 28 days. At the end of this phase, the concrete samples were subject to a drying phase (4 weeks at 60 °C) before being saturated under vacuum with a high-concentration sulfate solution (8.9 g/L sodium sulfate). This pre-saturation cycle, which lasted 48 h, can be considered a means of accelerating the test by reducing the time needed for a sufficient amount of sulfate ions to diffuse inside the sample. This step helped to overcome the kinetics of the penetration of sulfate ions linked to the physical resistance of concrete and was, therefore, an essential factor in the acceleration of the attack [58]. At the end of the pre-saturation phase, the concrete samples were directly immersed in the sulfate solution (8.9 g/L sodium sulfate) for a period of 12 weeks with solution renewal taking place once every month. For example, Figure 10 illustrates the expansion results obtained by Messad [58] for various concrete specimens (see [58] for details of the studied concrete mixes). From Figure 10, it can be observed that the level of expansion of concrete samples containing mineral additions (slag and pozzolans) was smaller than that of samples made with OPC.

#### 2.2.2. Method of the Institute of Research in Civil and Mechanical Engineering (GeM-France)

**Figure 11:**
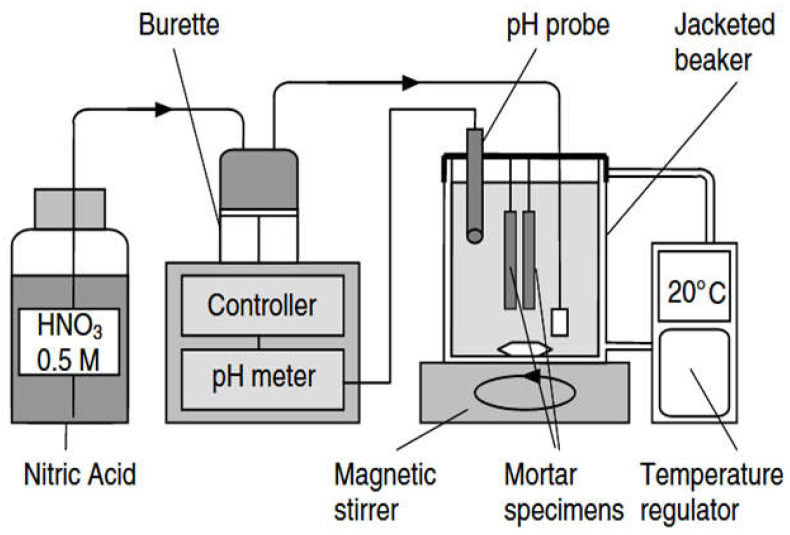
Schematic representation of the experimental setup used by El-Hachem et al. [93].

Most of the accelerated-aging methods developed for the study of ESA in cement paste, mortar or even concrete samples use high sulfate concentrations of the order of 10 g/L or more. This order is larger than that encountered in real life situations and on sites [93]. This high concentration leads to accelerated degradation, but also to some modifications of the mechanism of ESA. To overcome these difficulties, the Institute of Research in Civil and Mechanical Engineering (GeM, Nantes, France) developed an innovative method. This method was applied by El-Hachem et al. in [93]. In this study, the pH and temperature of the immersion solution were kept constant at 7.5 and 20 °C respectively. The pH regulation was carried out by adding nitric acid solution (0.5 mol/L). Figure 11 illustrates the experimental setup used in this method (see also [93,94]). The principle of this test-method consists of keeping the mortar specimens in a small tank containing sulfate solution at constant pH and concentration. These boundary conditions are meant to be representative of field conditions where cementitious materials are exposed to a continual supply of sulfate ions.

**Figure 12:**
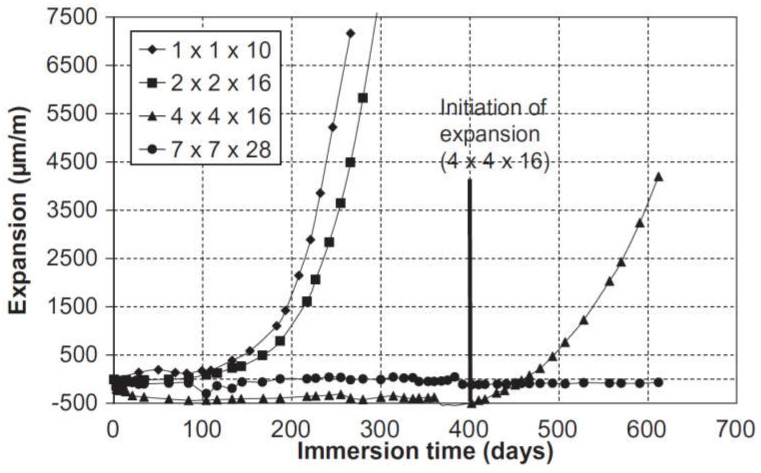
Expansion of mortar prisms of different sizes [93].

In the experimental program used by El-Hachem et al. [93], mortar prisms of different sizes (1 × 1 × 10 cm^3^; 2 × 2 × 16 cm^3^; 4 × 4 × 16 cm^3^ and 7 × 7 × 28 cm^3^) were tested to investigate the effect of size on their performance against ESA. The prisms were placed at 100% RH for 24 h and then left under limewater for 27 days. After the curing period, the mortar samples were immersed in a 3 g/L sodium sulfate solution. From the expansion measurements (see Figure 12), two phases of behavior were identified for all tested sizes (except for the 7 × 7 × 28 cm^3^ prisms where the expansion remained constant, even after 600 days of ESA). During the first phase (latency period), the expansion was stable and low. However, the second phase was characterized by an important increase in expansion for all samples. Interestingly, in the case of smaller prisms (1 × 1 × 10 cm^3^ and 2 × 2 × 16 cm^3^), the second phase started early (after 200 days of immersion in sodium sulfate solution) whereas significant expansion was found in the 4 × 4 × 16 cm^3^ prisms after 400 days of immersion [93].

**Figure 13:**
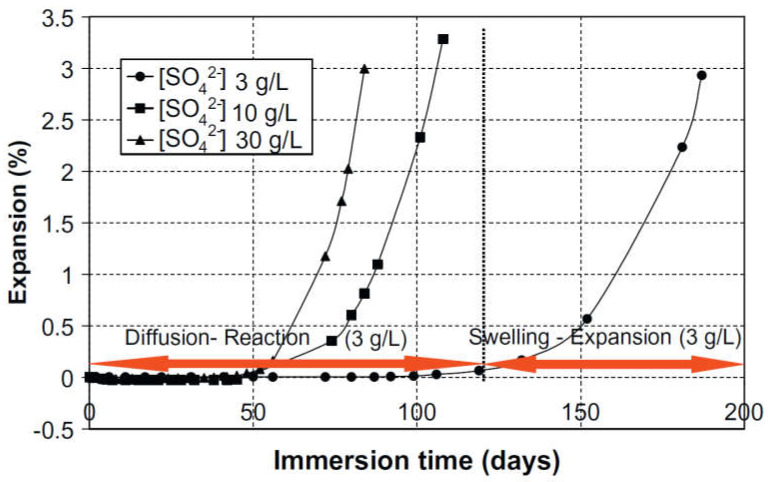
Expansion of mortar prisms (2 × 2 × 16 cm^3^) immersed in three different concentrations of sodium sulfate [93].

Moreover, the effect of the sulfate concentration of the immersion solution was evaluated by fully immersing mortar prisms (2 × 2 × 16 cm^3^) in three different sodium sulfate solutions with the following concentrations: 3 g/L, 10 g/L, and 30 g/L. The curing phase in limewater lasted for 15 days. The experimental results (see Figure 13) clearly showed that the response to ESA was very similar with the three concentrations. However, the onset of the second phase (significant expansion) was delayed when the samples were immersed in a low concentration solution (3 g/L of SO_4_^2−^) [93].

This experimental study validated that both the size of the sample and the concentration of the solution can influence the behavior of a cementitious material exposed to ESA. Based on the results, the exposure of small mortar samples to high concentrations of sodium sulfate leads to greater degradation within a short period of time.

#### 2.2.3. Method of the German Institute for Civil Engineering (DIBT-Germany)

In Germany, studies on effects of sulfate attack on cement-based materials are conducted by applying one of the three accelerating testing methods [95]: the SVA method [96], the Wittekindt [97] method, and the CEN [80] method. An overview of these techniques is proposed in Table 2, where it can be observed that the main common point between the SVA method and the Wittekindt method is the definition of a sulfate resistance criterion based on an expansion limit of 0.5 mm/m after a specified duration of exposure to sodium sulfate solution.

Mielich and Ottl [95] conducted an investigation on an existing structure (sports hall foundation in Germany) after the apparition of significant cracks in the floor. A possible exposure to ESA was then considered due to the increased amount of sulfate in the groundwater of the site. The SVA method [96] (i.e., testing method recommended by the committee of experts of the German Institute for Civil Engineering) was found to be more applicable in this case in order to compare the behavior of concrete samples extracted from the foundation of the damaged structure with concrete or mortar samples fabricated in the lab under controlled conditions.

**Figure 14:**
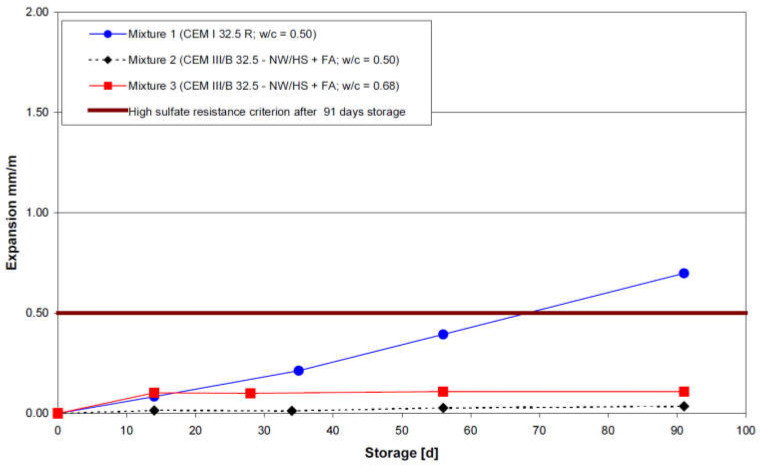
Expansion behavior of mortar prisms immersed in sodium sulfate solution (29.8 g/L) at 20 °C from [95].

The study conducted by Mielich and Ottl [95] was composed of two main parts. The first part of the experimental study was performed using flat mortar prisms (10 mm × 40 mm × 160 mm). Directly after fabrication, the mortar samples were stored in moist air for 48 h and 90% RH. After this initial storage period, demolded specimens were subject to a 12 day cure in saturated Ca(OH)_2_ solution, immediately followed by 91-days of immersion in high sodium sulfate solution (29.8 g/L). Prisms were fabricated with two cement types (CEM I/OPC and CEM III/B with 73.8% fly ash) and two w/c ratios (0.5 and 0.68). The higher w/c ratio was only used in the casting of CEM III/B prisms. Hence, three different mortar mixes were finally tested against accelerated sulfate attack (mixture 1: CEM I/OPC with w/c of 0.5; mixture 2: CEM III/B with w/c of 0.5 and mixture 3: CEM III/B with w/c of 0.68). The resistance of mortar samples was investigated by monitoring the expansion at the initial state (after cure in saturated Ca(OH)_2_ and therefore before any contact with the sodium sulfate solution) and during the accelerated attack. Expansions were measured at specific time intervals (14, 28, 56, and 91 days). Figure 14 shows the expansion behavior of mortar samples immersed in 29.8 g/L sodium sulfate solution at 20 °C. 

It can be observed that the expansion of mixture 2 and mixture 3 samples (both made with CEM III/B) did not exceed the recommended criterion value of 0.5 mm/m at the end of the exposure period. However, mixture 1 containing CEM I showed significant expansion after 91 days of immersion in the sodium sulfate solution that exceeded the recommended threshold limit. According to Mielich and Ottl [95] these observations were expected, since CEM III/B cement is considered to be sulfate resistant in the German specifications [96].

**Figure 15:**
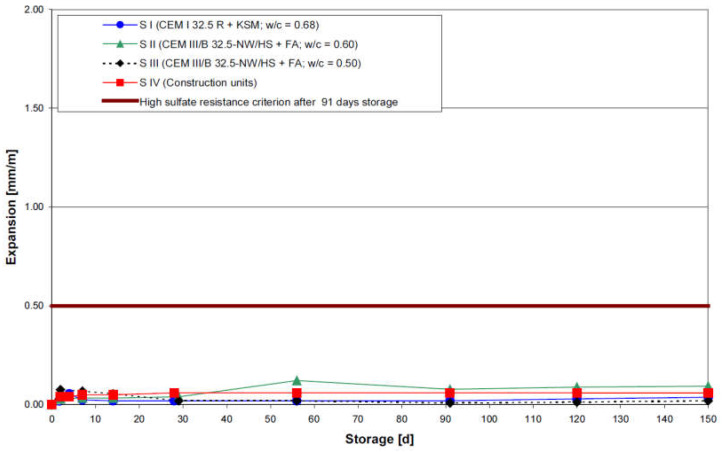
Expansion behavior of concrete drill-out cores immersed in sodium sulfate solution (29.8 g/L) at 6 °C from [95].

**Figure 16:**
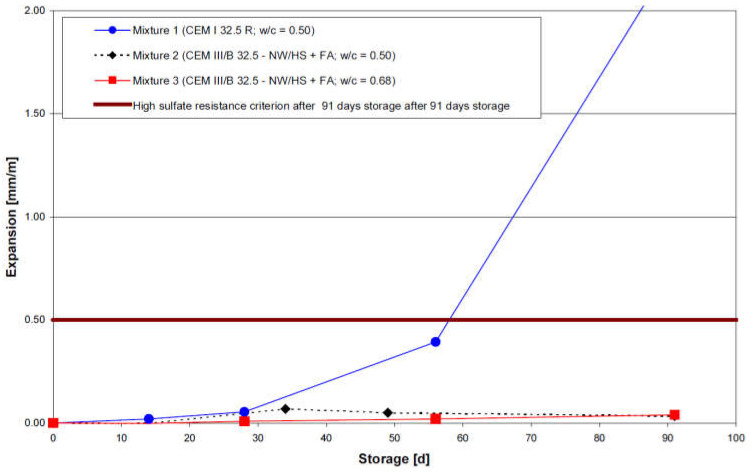
Expansion behavior of concrete drill-out cores immersed in sodium sulfate solution (29.8 g/L) at 20 °C from [95].

In the second part of the study, concrete cores with a diameter of 50 mm that were extracted from the foundation of a damaged structure were tested. The concrete of the building was produced with CEM III/B containing 50% fly ash and with a high w/c ratio of 0.68 (this concrete mixture is labeled S IV in the following). To produce cored companion samples, 200 mm^3^ cubes made of three different concrete mixtures (S I: CEM I with w/c of 0.68; S II: CEM III/B with w/c of 0.6 and S III: CEM III/B with w/c of 0.5) were cast and kept in a moist room at 20 °C and 95% of RH for two days. After 48 h, cores with a 50 mm diameter were extracted from these lab-manufactured specimens, sawed (at 150 mm length) and polished at the top in order to resemble the site cores as much as possible. Before immersion in the sodium sulfate solution (29.8 g/L) at two different temperatures (20 °C and 6 °C), all concrete specimens (extracted from the site and from cubes) were stored for 12 consecutive days at 20 °C and 65% RH. A temperature of 6 °C was considered in order to investigate the thaumasite form of sulfate attack. The expansions measured during the sulfate attack are shown in Figure 15 (immersion at 6 °C) and Figure 16 (immersion at 20 °C).

**Figure 17:**
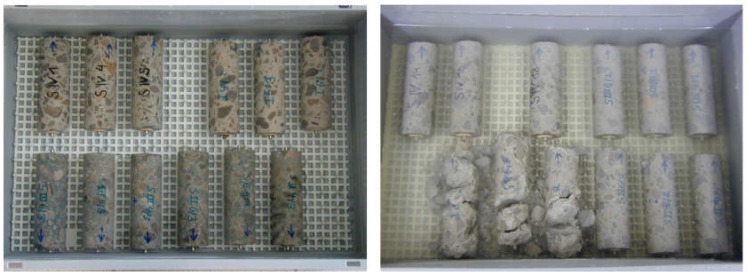
Concrete drill-out cores of type S I at the beginning of the test (**left**) and after 150 days of immersion in sodium sulfate solution (29.8 g/L) at 6 °C (**right**) [95].

From Figure 16, it can be seen that no samples immersed at 20 °C reached the expansion limit (0.5 mm/m). On the other hand, the expansion measurements recorded from samples immersed at 6 °C showed that concrete samples made with CEM I (S I) exceeded the allowed criterion after 91 days. Although the remaining samples did not reach the limit, it should be noted that significant expansion was observed in the S III samples. The aging process continued for sample S I and S IV with exposure to sulfate for an additional period of 390 days (not presented in Figure 15 and Figure 16). The results showed significant expansion for sample type S I at 20 °C with 3.46 mm/m after 540 days, whereas for S IV, the expansion rate remained low and barely reached 0.06 mm/m. In addition, a visual observation was conducted for S I samples after 150 days of immersion in sodium sulfate solution at 6 °C (see Figure 17). The visual deterioration was attributed to the formation of thaumasite resulting from the presence of sulfate at low temperatures (6 °C). However, it must be underlined that the high sodium sulfate solution concentration involved in the testing protocol (29.8 g/L) may have induced significant changes in sulfate attack mechanisms, leading to questionable conclusions regarding the real sulfate resistance level of the tested material.

#### 2.2.4. Electrical Pulses Acceleration Method

**Figure 18:**
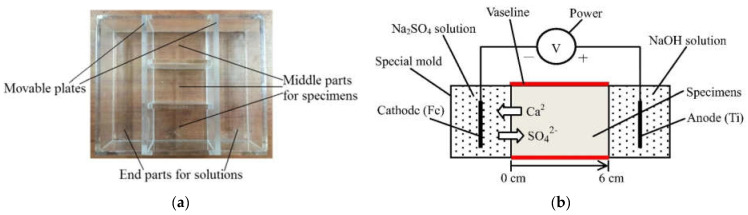
Accelerated sulfate attack test developed by Huang et al. [101]: (**a**) The mold used during the combined sulfate attack (image from [101]); (**b**) Schematic representation of the combined sulfate attack (image from [101]).

Inspired by the usual method used for accelerating the penetration of chloride ions in concrete [98,99,100], a new method of accelerating the sulfate ingress in mortar samples was developed by Huang et al. [101]. In this technique, an electrical field produced by electrical pulses is used to increase the rate of sulfate ion migration inside a cement-based material, hence accelerating the ESA. In the experimental protocol proposed by Huang et al. [101] during their research work at the College of Materials Science and Engineering in the university of Chongqing (China), prismatic mortar samples (40 × 40 × 60 mm^3^) made from various types of cement (PC: CEM I, LP-30: CEM I with 30% Limestone powder and FA-30: CEM I with 30% fly ash) were cast into special molds containing, in the center part, three prismatic locations for mortar with two containers on the sides for the sulfate solution (as shown in Figure 18a). After 24 h, the movable parts of the mold were pulled out, and the mortar samples were cured for 27 days at 20 °C and 95% RH. Twenty-seven days later, the cathodic compartment was filled with 5% sodium sulfate (Na_2_SO_4_) solution, while a 2.8% NaOH solution filled the anodic part of the testing mold. One of the surfaces of the specimen was exposed to sulfate solution (40 mm × 40 mm), while the remaining five surfaces were sealed with Vaseline (see Figure 18). A low-frequency electrical pulse was applied (30 V cyclically applied for 20 s followed by 20 s of rest). Thus, the surface of the tested sample closer to the sulfate solution was exposed to a combined attack (immersion and electrical pulse). 

**Figure 19:**
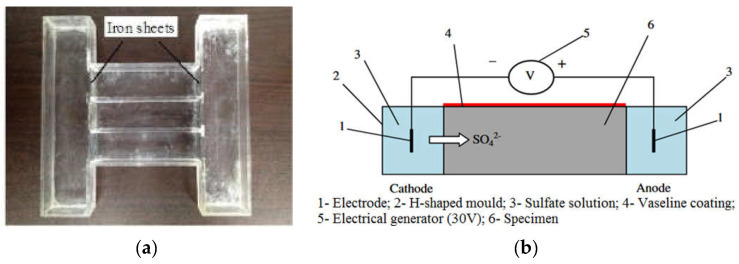
Accelerated tests conducted by Huang et al. [102]: (**a**) The H-shaped mold used for the electrical pulse test; (**b**) Representation of the different parts of the electrical pulse test.

**Figure 20:**
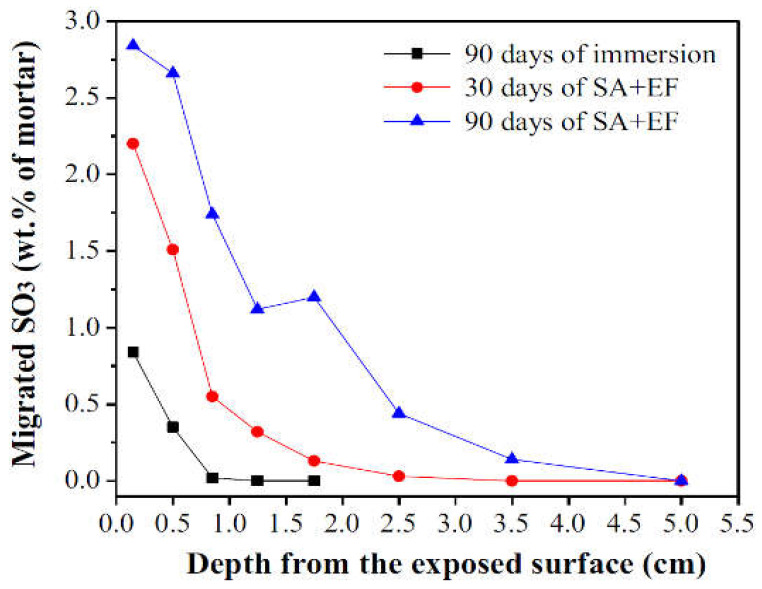
Migrated SO_3_ content in PC mortar samples as a function of the depth from the exposed surface [101].

In another experimental program, the same group of researchers applied [102] a modified protocol to perform an electrical pulse accelerated sulfate attack. In this study, the considered prismatic mortar samples were longer than previous ones (40 mm × 40 mm × 160 mm), and only a sodium sulfate solution was used, whereas two solutions (sodium sulfate solution and NaOH solution) were needed to carry out the previous testing protocol. In this second experiment, samples were cast in the middle part of H-shaped molds where the accelerated test was run (Figure 19a). After casting, the samples, together with the molds, were stored in climatic chambers at 20 °C and 98% RH. Twenty-four hours later, the iron sheets shown in Figure 19a fixed at both edges of the mortar samples were removed before returning the specimens to the moist room for 27 days of curing. At the end of this phase, the upper part of the mortar samples was coated with Vaseline to protect against any type of damage or corrosion. Hence, the accelerated attack by electrical impulsions was launched by filling the two containers of molds with sulfate solution (33.8 g/L of sodium sulfate) and installing two titanium electrodes at both the cathodic and anodic compartments. The pulsed electric field was generated by applying a low-frequency electrical pulse with a voltage of 30 V for 20 s followed by another 20 s of rest [102]. The accelerated sulfate attack lasted for 90 days. Whether the mold was H-shaped or a square, the same approach was used in order to accelerate the ESA and then evaluate the sulfate resistance of the mortar samples. In both cases, the study included a series of measurements in order to evaluate the consequences of electrical pulses during the sulfate attack. For example, the sulfate concentration corresponding to the SO_3_ content in mortar samples was measured according to Chinese standard GB/T 2008 [103] by chemical titration (barium gravimetric method). Figure 20 illustrates the migrated sulfate content of samples made from CEM I immersed in the sulfate solution or subjected to combined exposures of immersion and electrical pulses (SA + EF in the figure). The results show an SO_3_ content of 0.85% at a depth of 0.2 cm after 90 days of immersion. However, the combined exposure led to greater SO_3_ distribution (2.25%) and (2.7%) at a depth of 0.2 cm after 30 days of SA + EF and 90 days of SA + EF, respectively [101]. These results show that the electrical pulse method can elevate the rate of sulfate migration during ESA when combined with conventional attack by full immersion.

In addition, the mechanical properties of the attacked samples (compressive and flexural strengths) were evaluated. To identify the changes in the microstructure and the mineral phases at the end of the accelerated attack, microscopic observations were carried out using a scanning electron microscope and X-ray diffraction techniques. In conclusion, it was observed that accelerating ESA via the electrical pulse technique helped, not only by increasing the migration of sulfate ions into the samples but also by accelerating the leaching process which led to a complete dissolution of portlandite (CH), followed by a decomposition of C-S-H. The overall increase in the amount of sulfate diffused into the mortar samples induced severe deterioration and damage by the formation of both ettringite and gypsum. This was confirmed by the mechanical strength losses observed after the attack. These results show that this accelerating technique, if properly applied, can be considered a fast method that can be used to study the effects of ESA on cement-based materials [101,102,104].

## 3. Discussion

As previously discussed, several methods are used to determine the sulfate resistance of cement-based materials. Most test methods involve full or partial immersion of mortar, concrete, or cement paste in a sulfate solution with a specific concentration and with or without pH regulation [27,30,46,47]. Others have involved the use of continuous wetting and drying cycles to simulate marine conditions, and especially the damage induced by salt crystallization [6,34,50,51,52,53,75]. There are several study strategies and resistance criteria related to ESA. For example, the US methods more directly study the relationship between the strength of mortar exposed to ESA and its expansion. However, in some Chinese studies, sulfate attack was accelerated by electrical pulses and sulfate resistance was assessed based on the loss of strength [77,101,102]. The European approach requires continuous monitoring of the expansion and microstructural changes of samples exposed to a sulfate solution [17,76,93,105,106]. ESA studies based on expansion measurements such as the ASTM C 1012 test method and the European test method have been conducted since the 1930s. However, many studies and reports have shown that different erosive sulfate solutions as well as various physical parameters can be considered to test and qualify sulfate resistance. Indeed, the concentration of the sulfate solution has a direct effect on the kinetics and mechanisms associated with the attack [8,78,93]. For example, high sulfate concentrations (up to 30 g/L) are mostly associated with the gypsum formation rather than the AFt phase [78]. However, exposure to lower concentrations (3 g/L), usually found in the field, suggests a different scenario, because gypsum is difficult to stabilize under such conditions and, consequently, ettringite will be the main compound produced during the attack [45,107,108,109,110,111,112]. On the other hand, the frequency of the solution renewal is another factor influencing the ESA mechanism because it has been shown that sulfate penetration is accelerated by continuous renewal of the solution (every 15 days or every 30 days) [113].

The pH of sulfate solution can significantly affect the progress of ESA. The leaching process that occurs during erosion and the dissolution of the cement paste phases directly affects the pH of the solution. Some studies recommend the use of pH regulation while others prefer to maintain uncontrolled pH. For example, the method developed by the GeM [93], which, requires the pH values to be stabilized, has been applied in many cases using a circulating solution to keep the pH constant by manual titration with H_2_SO_4_ [114]. Moreover, Mehta et al. [31,115] tried to mechanize pH regulation by using continuous titration with H_2_SO_4_, while monitoring the pH using a pH controller. During their experimental work, the surrounding sulfate solution was purely acidic stabilizing the pH at 6.2. The results showed that, under such exposure conditions, the degradation process was mainly dominated by gypsum formation. Moreover, it has been reported that, under these conditions, the low C_3_A content did not improve the resistance against sulfate ingress because negligible amounts of ettringite are formed during ESA compared to gypsum [31,115].

In [29,30], the authors used a similar approach but with three different values of constant pH (6, 10 and 11.5). It was stated that by keeping the pH of the sulfate solution stable, the test becomes more representative of the field conditions as well as more accelerated since the expansion of the samples occurs faster than that of specimens placed inside a typical non-controlled pH sulfate solution (coupling between the sulfate attack and the cement leaching). According to [29,30], the resistance of mortars to ESA decreases when the pH of the erosive solution is below 10. This conclusion was confirmed by Revertegat et al. [116] while testing the performance of cement paste samples. These authors mentioned that the resistance to sulfate solutions reduces for pH values lower than 11.5 and this reduction is common for samples made with Portland cement (CEM I) and cement containing slag (CEM III). The degradation observed in materials exposed to sulfate solutions with constant pH at low values is believed to be caused by the increase in porosity, significant calcium (Ca^2+^) leaching and crack formation at the surface level [116]. However, these symptoms are not observed with samples exposed to solutions with non-stabilized pH where the attack is hugely dominated by the penetration of sulfate ions through the cement matrix [116].

Studies on the effects of the specimen size and/or shape have shown that a higher expansion rate with smaller prisms (10 × 10 × 100 mm^3^), leads to faster results compared to the standard 25 × 25 × 285 mm^3^ specimens [16,76,105,117]. The expansion measurements used in the ASTM C 1012/C 1012M-18 testing method [70] were carried out on large prisms (25.4 × 25.4 × 25.4 mm^3^) placed in contact with a high concentration sodium sulfate solution (50 g/L). In a study by Ferraris et al. [105], the conditions recommended by ASTM C 1012 were replicated, but with smaller samples (10 × 10 × 40 mm^3^). Interestingly, the same level of degradation is achieved but in a shorter time. Based on this, Ferraris et al. stated that the use of small samples could reduce the time needed to observe critical damage caused by ESA.

In the same study, Ferraris et al. tried to cover not only the effect of size but also the effect of shape (prism or cylinder). They compared the expansion evolution between prisms (25 × 25 × 279 mm^3^) and cylinders with varying diameters (25 mm, 50 mm and 75 mm) and lengths of 152 mm. The results were almost similar for both geometries, confirming the importance of size over shape (geometry) when accelerating ESA.

Conventional testing methods of ESA are more closely related to the ettringite form of the sulfate attack on the cement aluminates. However, these methods do not take into account the conditions in which the concrete is exposed to salt crystallization pressures (evaporative transport) or to the low temperatures which enhance the formation of thaumasite by attacking the C-S-H [17,105,106,114]. A brief summary of the existing test methods and standards, including those previously described, is shown in Table 3.

Most laboratory tests provide a chronological evaluation of the samples. However, the deterioration state can be immediate in field tests, and this can be related to the difficulty of controlling the exposure conditions, such as the temperature and relative humidity, in the field. The comparison between exposure to sulfate under controlled laboratory conditions and in the field has shown good correlations in terms of distress and failure. However, the possibility of reproducing the technology used in the ESA study and transferring it to a construction project is uncertain. Concrete quality assessment methods at real construction sites primarily focus on non-destructive testing to characterize the structure in a particular condition. With all the problems and complications associated with ESA it is doubtful whether a single test can solve all these problems. In addition, investigating all potential exposures and associated conditions is extremely expensive and time-consuming. To overcome these difficulties, several authors have performed thermodynamic calculations with the help of specific software to model and predict the progress of the ESA by taking into consideration several materials placed in different erosive solutions at different temperatures [22,23,27,32,118].

Many researchers believe that most of the test methods listed in literature evaluate the exposure of mortar or paste samples to sodium sulfate solutions within unreasonable time frames [93]. However, lab-based investigations can be considered consistent given the complex mechanisms and processes associated with in situ ESA. In addition, tests conducted under controlled laboratory conditions take many parameters (cement type, type of cation, sulfate concentration, quality and type of concrete, exposure conditions, type of accelerated attack, etc.) into account that should be implemented when testing the protocol and then analyzed in order to predict the level of damage under all possible circumstances and perspectives [70,74]. Moreover, the various combinations of different attack mechanisms observed during ESA (chemical/physical interactions) as well as the importance of representing the field conditions make it extremely difficult to develop a single and appropriate test method. In this context, a complete approach that includes the laboratory and field performances of cement-based materials exposed to various sulfate environments and exposure conditions is needed to obtain a reasonably comprehensible and adequate representation of the impact of ESA on the durability of a concrete structure.

The accelerated ESA protocol intentionally induces microcracks in the experimental material resulting in greater expansion due to specific sulfate concentration and pH regulation (LMDC and GeM methods), and has been shown to be effective, but time consuming. The methods developed in Europe, when compared to the US methods recommended by ASTM or USBR, are considered more effective in the long term with a significant increase in the rate of sulfate ingress, resulting in significantly faster rates of expansion and deterioration [57,119].

Accelerated tests on mortar samples were proven to be significantly shorter in time compared with tests performed on concrete specimens. This is mainly attributed to the specimen size, its permeability, and the cement content. However, using concrete with a relatively high w/c ratio was associated with significant improvements, obtaining results over a shorter period depending on the type of mixture and mineral additions [116,120,121,122,123]. The results for methods developed by accelerating the attack with electrical pulses [101,102] show that severe damage and strength loss were obtained in a short period of time. This technique, which has been applied on mortar samples, proves that the traditional immersion in sulfate solution is not the only way to study sulfate resistance. However, the significant migration of sulfate and hydroxide anions during the electrical pulse method results in further dissolution of portlandite, which can vary the pH of the solution and cause more calcium leaching. This entire process should be considered and discussed when applying electrical pulses to accelerate ESA [101,102].

Several attempts have been made to develop a test method that can quantify the distress and deterioration levels caused by ESA in construction projects. However, it should be noted that the kinetics of the in situ attack are much slower than in accelerated tests, so it may take several years to see serious deteriorations of the investigated structure. This raises concerns about the possibility of finding a way to analyze the entire attack mechanism. Additionally, the application of a testing method to existing projects or structures requires several field inspections and appropriately developed techniques, which can be complex and expensive for construction firms. Therefore, the results obtained in laboratory tests are considered crucial to determine the possible results and consequences of exposure of concrete structures to ESA [124]. Accelerated ESA tests applied on cement pastes and mortar samples are not directly comparable to tests involving concrete. Cohen and Mather [74] stated that the ITZ (interface between the cement paste and an aggregate) interferes in the transport process that occurs in concrete, since this zone has a significant porosity level. The presence of ITZ, consequently leads to a different degradation mechanism and damage level caused in concrete than that observed in cement pastes and mortars. Furthermore, İnan et al. [125] indicated that, in the case of concrete exposure to ESA, the presence of an interface between cement and aggregates allows for more ettringite precipitation, especially in the case of CEM I. This process can help with the analysis of the mechanism of expansion caused by ettringite formation during ESA, which might not correspond to the ESA mechanisms developed in cement pastes and mortars. 

Based on this, results on concrete are generally considered more representative of the cases commonly seen in fields [125]. It should be noted that the experimental approaches used to accelerate the attack (pH control and high sulfate concentrations) can be applied indiscriminately to all material types (cement paste, mortar or concrete) while the consequences and the level of distress can differ [125].

Each experimental program devoted to the study of ESA has different strengths and weaknesses. Some researchers believe that the use of higher sulfate concentrations is the most efficient way to achieve adequate results in an acceptable amount of time. However, others state that this technique can only approximate the natural process of the attack. Concerning the shape (cylindrical or prismatic) and the type (concrete, mortar or cement paste) of samples, the debate is always framed in terms of the reliability and representativeness. This highlights the need to unify all these propositions in a common experimental approach. However, the difficulties associated with ESA make any future work subject to many debatable interpretations. Lastly, it is recommended that further studies should be conducted in order to develop well-established scientific methods that are directly related to real-life conditions and exposure sites, with the definitive goal of benchmarking accelerated controlled laboratory tests. Combining predictive models and software calculations with rapid metric testing will ultimately yield useful solutions, but further research is needed to implement them into standards and building codes.

An appropriate test method for studying ESA resistance should be reliable and reproducible, but also rapid, relevant and practical, and should not exceed expectations. However, it should be considered that although tests may address certain performance and durability issues for general industrial or scientific issues, they should relate to actual and situational conditions as closely as possible. This is not the case for most external sulfate resistance testing methods which only measure the resistance of a cementitious binder (typically in a mortar sample) when fully immersed in a sulfate solution (like ASTM standardized methods [41,70]). In addition, due to the complicated aspects related to ESA, a representative testing method must take into consideration the cation type effect (calcium, sodium or magnesium). Moreover, the ESA encountered in marine conditions involves chloride ions in addition to the sulfate attack, which makes the mechanism very complex. In marine environments, there is a double effect of both chlorides and sulfate salts, which can affect the durability of reinforced concrete structures due to the combined action of corrosion and ESA. Free chlorides initiate steel corrosion while sulfates damage the structure by expansion [126]. According to some authors (e.g., see [127]), chloride ions (Cl^-^) are partially absorbed by calcium silicate hydrate (C-S-H) and can connect to monosulfoaluminate to form Friedel’s salts or hydrocalumite (3CaO Al_2_O_3_ CaCl_2_ 10H_2_O) which can decrease the effects of ESA by limiting the stability of ettringite. Due to the complexity of the process, the penetration of chlorides and sulfates into cement-based material exposed to marine environments is studied independently.

Table 4 synthetizes the recommendations suggested to enhance the test-methods.

## 4. Conclusions

Sulfate attack is a widespread form of physical–chemical attack on concrete physical-chemical attack and has always been an important part of research on the durability of concrete structures. Although various testing methods for resistance to sulfate attack have been proposed, so far each method has certain advantages and disadvantages which should be considered in terms of ease of implementation and cost representativeness. With certain limitations, no method can quickly and accurately reveal the mechanism associated with concrete sulfate attack, especially for the long-term prediction of various properties of concrete in sulfate environments. It is therefore particularly important to continue research efforts to develop optimized sulfate attack test methods.

In this paper the main methods of evaluating mortar and concrete resistance to sulfate attack are reviewed. Some limitations of these methods are mentioned below:

- The ASTM C 452-15 can result in the sulfate attack of anhydrous compounds, and the presence of gypsum can cause sulfate attack in the fresh state. This early attack means that the testing conditions of this method are not similar to field exposure.
- The ASTM C 1012 is a much longer test method (about 18 months) compared to the ASTM C 452, and it is not considered to be the best or the most connected to real-life exposure conditions.
- The USBR 4908 method is also a much longer test as the test period varies between 1 and 2 years. This method does not support the continuous development of new sulfate-resistant cements.
- The Swiss standard SN 505262/1 can provide relevant information about the resistance of concrete against ESA. However, the exposure conditions of the specimens are not similar to the field conditions (consecutive cycles of drying–wetting followed by full immersion of the specimens).
- The Canadian standard CSA A3004-13 is very similar to the ASTM C 1012 method, but it considers the formation of ettringite and thaumasite forms of the sulfate attack. This method uses a very highly concentrated sulfate solution that is not consistent with the field conditions.
- The Asian standard GB 749-1965 requires a long test period without any information about the sulfate solution concentrations. Even though the duration is shorter than that of other proposed standards (GB/T 2420-1981, GB/T 749-2001 and GB/T 749-2008 for example), this method has a major disadvantage in that the size of the test specimen is too small, and no complementary tests have been proposed to explain the test results.

Non-standardized methods were also proposed in this paper. All of them are satisfactory but could conceivably benefit from improvements (Table 4). 

The continued research and efforts in the field of sulfate attacks have aided in the exploration of new techniques (high sulfate concentrations, small test specimens, electrical pulses, etc.) that could be used to study ESA over short periods of time. However, many researchers believe that these methods modify the ESA mechanisms encountered in reality. In this context, another question may arise in terms of the ability of these accelerated techniques to reflect realistic and achievable conclusions regarding ESA resistance. When a cementitious material in the field is exposed to a sulfate-rich environment, it would be of paramount interest to test the resistance of its composition beforehand. In addition, important factors related to the type of sulfate environment, sulfate solubility, and concentration should be determined and evaluated during the experimental approach. For example, having both sodium and magnesium sulfates makes the mechanism of ESA more complicated, and in this case, the traditional experimental approaches of immersion in a sodium sulfate solution (US and European methods) become inefficient for studying the effects of the coupling between sodium and magnesium. Moreover, thaumasite formation and salt crystallization are two independent types of attack that require the use of different experimental approaches to analyze the damage process. 

## Figures and Tables

**Table 1:** Experimental exposure conditions of the accelerated sulfate attack proposed by Messad [58].

Parameters of the Exposure Conditions	
Type of exposure	Immersion
pH	7 by H_2_SO_4_ solution
Sulfate concentration	8.9 g/L of Na_2_SO_4_/6 g of SO_4_
Temperature	25 °C
Solution renewing	Each 4 weeks
Preconditioning	Saturation under vacuum by Na_2_SO_4_ at 8.9 g/L
Test duration	12 weeks

**Table 2:** Comparison of German accelerated test methods and corresponding criteria based on expansion measurement from [95].

	Wittekindt [97]	SVA [96]	CEN [80]
Specimen geometry	10 × 40 × 160 mm^3^	10 × 40 × 160 mm^3^	20 × 20 × 160 mm^3^
w/c ratio	0.60	0.50	0.50
Sulfate solution (Na_2_SO_4_)	4.4% (29,800 mg sulfate/L)	4.4% (29,800 mg sulfate/L)	2.4% (16,000 mg sulfate/L)
Storage period	56 days	91 days	not defined
Criterion	≤0.50 mm/m	≤0.50 mm/m	not defined

**Table 3:** Review of some ESA test methods.

Testing Method	Specimens Size	Curing	Method of Attack	Property Determined	Weaknesses
ASTM C 452	Mortar prisms (25 × 25 × 285 mm^3^)	Moist closet for 23 h followed by storage in water for 30 min, at 23 °C	In fresh water at 23 °C; replenish water every 7 days	Length change compared to nominal gage length after 14 days	Not similar to the field exposure
ASTM C 1012	Mortar prisms (2 × 25 × 285 mm^3^)	Seal in mold immersed 23 h in water at 35 °C followed with lime water at 23 °C	5% sodium sulfate solution at 23 °C; pH = 6–8	Expansion, mass variation	Long duration of test
USBR 4908	Concrete cylinders (d = 150 mm; h = 300 mm)		Method A: immersion in 14 g/L Na_2_SO_4_; Method B: immersion in 68 g/L Na_2_SO_4_; Method C: alternate immersion in 14 g/L Na_2_SO_4_ for 16 h and wet/dry cycles for 8 h at 54 °C	Expansion, mass variation	Long duration of test
LMDC	Concrete cylinders (d = 110 mm; h = 220 mm)	Pre-saturation for 48 h in a 8.9 g/L Na_2_SO_4_ solution	Immersion in a 8.9 g/L Na_2_SO_4_ solution at 25 °C, pH = 7, duration of the test 12 weeks	Expansion, mass variation, compressive strength	Not similar to the field exposure
GeM	Concrete prisms (70 × 70 × 280 mm^3^) and (110 × 110 × 220 mm^3^) Mortar prisms (40 × 40 × 160 mm^3^) and (20 × 20 × 160 mm^3^) and (10 × 10 × 100 mm^3^)	Storage at 20 °C and 50% RH followed by drying at 40 °C	Immersion in [SO_4_^2−^] = 3 g/L, [SO_4_^2−^] = 10 g/L and [SO_4_^2−^] = 30 g/L; pH = 7 at 20 °C	Expansion, mass variation, compressive strength	Not similar to the field exposure
Swiss Method	Concrete prisms (150 × 150 × 150 mm^3^)	Storage at 20 °C and 90% RH	Drying at 50 °C for 120 h followed by immersion in a 5% sodium sulfate solution	Expansion, mass variation, compressive strength	Not similar to the field exposure
German method	Mortar prisms (10 × 40 × 160 mm^3^) Concrete drill-out cores (𝝓 50 mm)	12 days in saturated Ca(OH)_2_ 12 days at 20 °C and 65% RH	Immersion in 28.9 g/L sodium sulfate solution for 91 days	Expansion measurements	Not similar to the field exposure
Asian method	Mortar prisms (40 × 40 × 40 mm^3^) and (40 × 40 × 160 mm^3^)	Storage at 20 °C and 95% RH	Combined attack (immersion in 5% Na_2_SO_4_ and low frequency pulsed electrical field)	SO_3_ profiles, compressive strength	Long duration of test
CSA A 3004-C8	Mortar prisms (25 × 25 × 285 mm^3^)	Drying at 38 °C for 24 h followed by 24 h immersion in lime water	Sulfate sodium solution (50 g/L) at 23 °C	Expansion, mass variation	Not similar to the field exposure
CSA B 3004-C8	Mortar prisms (25 × 25 × 285 mm^3^)	Drying at 38 °C for 24 h followed by 24 h immersion in lime water	Sulfate sodium solution (50 g/L) at 5 °C	Expansion, mass variation	Not similar to the field exposure

**Table 4:** Recommendations for enhancing the existing test-methods.

Parameter	Recommendations
pH	Maintaining the pH of sulfate solution at a neutral value to increase the progress of ESA. This potential increase is due to the leaching process as well as the growth of the solubility of the cement paste phases
Sulfate concentration	The use of an intermediate sulfate concentration (about 10 g/L) in order to increase the ESA without significantly changing the mechanisms of this phenomenon
Solution renewing	The increase in the solution renewal frequency to increase the ESA damage.
Exposure condition	The use of drying/wetting cycles can increase the damage due to ESA. This type of exposure conditions enhances the permeability of the samples and consequently accelerates the sulfate ions ingress inside the material
Sample pretreatment	The test acceleration requires a specific pretreatment. Samples can be pretreated by sulfate saturation under vacuum before exposure to the given conditions
Sample size	The use of samples with a small size and/or shape can raise the expansion due to ESA
Coupling with other ions	The use of sulfate coupled to chloride can decrease the effects of ESA by limiting the stability of ettringite
Cation type	The representative testing method must take into consideration the cation type effect (calcium, sodium or magnesium)
Electrical pulse	The electrical pulse can increase the penetration of the sulfate ions inside the cement matrix
Sulfate solubility	The increase in sulfate solubility allows raising the amount of sulfate able to react with the cement constituents. This can be due by increasing the temperature of the sulfate solution
Temperature	The temperature can increase the sulfate solubility. However, the temperature value has to not exceed a threshold value for which ettringite becomes unstable
Type of materials	The performing of tests on mortar instead of concrete can significantly shorten the duration of ESA study, due to the lower specimen size, higher permeability and higher cement content of mortar samples
w/c ratio	The increase in w/c ratio could reduce the test duration
Solution/sample ratio	The volume of the solution must be more than ten times the volume of samples

## Data Availability

Not applicable.
